# Effect of chemical and biological additives on production of biogas from coffee pulp silage

**DOI:** 10.1038/s41598-023-39163-w

**Published:** 2023-07-27

**Authors:** Mengizam Tsegaye Getachew, Andualem Mekonnen Hiruy, Majid Mohiuddin Mazharuddin, Tadios Tesfaye Mamo, Temesgen Aragaw Feseha, Yedilfana Setarge Mekonnen

**Affiliations:** 1 Center for Environmental Science, College of Natural and Computational Sciences, Addis Ababa University, Addis Ababa, Ethiopia; 2 Institute of Atomic and Molecular Sciences, Academia Sinica, Taipei, Taiwan; 3 Nano Science and Technology Program, Taiwan International Graduate Program, Academia Sinica, Taipei, 11529 Taiwan; 4 Institute of Physics, Academia Sinica, Taipei, 11529 Taiwan; 5 Department of Chemistry, National Taiwan University, Taipei, 10617 Taiwan

**Keywords:** Energy harvesting, Renewable energy

## Abstract

Energy is the foundation of the global economy and is essential to human survival. Nevertheless, more than 60% of it comes from fossil fuels. That is not a replenished and scarce source. However, a sizable amount of organic waste is generated every minute throughout the world and can be used as a raw material to produce renewable energy. Among them, Coffee processing generates a huge amount of solid and liquid waste that is organic and can serve as raw material for biofuel production. Since coffee beans and powder are Ethiopia’s main exports, coffee pulp is easily accessible. Therefore, the main goal of this project is to convert this waste, which largely consists of organic materials, into a valuable product called Methane. The purity and yield of methane productivity are significantly influenced by the type of additives we use. This work systematically investigates the effect of chemical and biological additives on the productivity and purity of the Biogas from the coffee pulp silage in batch systems under mesophilic temperature (38 °C) for different ensiling periods and additive proportions. The chemical additives recorded the maximum biogas production (2980 ml) at an ensiling period of 40 days with high purity of about 70% biogas. The minimum Biogas was recorded at the ensiling period of 10 days by the control (T1) treatments, which was 634 ml. This work proves that biological additives produced the highest quality and quantity of Biogas from coffee silage.

## Introduction

Coffee (*Coffea Arabica L*.) is the most marketable crop plant. It is the most prized commodity and a hot drink that is enjoyed all around the world. In the majority of tropical nations, coffee is crucial to the economy^1–4^. Among them, Ethiopia is one of the leading exporters. Coffee fruits can be processed using either wet or dry methods^5–7^. To get at the seeds (beans), the dried cherry coffee’s exocarp, mesocarp, and endocarp must be removed by hulling, which results in a husk. During the wet processing, the coffee fruits are pulped, fermented, washed, and dried to yield husk and pulp^2,8–10^. Coffee pulp is the main solid residue from the wet processing of coffee barriers, and its constituents are approximately 41% of the wet weight of the coffee berry. In 1996, the world production of coffee wastes was estimated at around 22 million metric tons of coffee pulp, 2.4 million metric tons of mucilage, and 8.6 million metric tons of hulls^4,9,11,12^.

The production of energy from a waste source like coffee pulp silage is the main strategic tool for the sustainable development of societies. It ensures a clean energy supply and prevents the Environment from Greenhouse Gas emissions (GHG)^13–16^. Biogas production is one of the great economic important processes that control the emission of Methane (CH_4_) into the atmosphere. Methane influences the Greenhouse Effect 23 times more than CO_2_ and remains in the atmosphere for 12 years. Therefore, capturing Methane is the best way to prevent Global Warming because the Methane produced in biogas production is neutral and does not affect the environment^4,17–20^.

The main objective of this study was to evaluate the effect of biological and chemical additives on the quality of coffee pulp silage for biogas production at different silage preparation periods. We characterized the chemical and physical properties of coffee pulp and its Silage in biogas production parameters. We determined the coffee pulp composition in terms of its mineral contents. Moreover, we compared the quality and quantity of the Silage for biogas production at different ensiling periods.

## Materials and methods

### Chemicals

Analytical-grade chemicals and solvents were used in the study. The chemicals used were sulfuric acid, potassium hydroxide, boric acid, calcium carbonate, copper sulfate, hydrochloric acid, nitric acid, perchloric acid, sodium hydroxide, petroleum ether, 1.25% H_2_SO_4_, 1.25% NaOH, homo fermentative lactic acid bacteria, lithium meta borate, lithium tetra borate.

### Materials and instruments

The apparatus and materials used in the study were glass bottle with lids, beaker, volumetric flask, Erlenmeyer flask, iron wire, plastic bags, funnel, deep freezer, pH meter, analytical balance, stirrer, desiccators, crucible, hot plate, gas kit maker, magnetic stirrer, incubator, oven, furnace, and instruments such as flame atomic absorption spectrophotometer, gas analyzer, water bath, and chopping. In the present study, different additives were used for silage preparation; such additives were biological and chemical^5,21,22^. The biological additives were the combined powder form of homo fermentative with heterofermentative lactic acid bacteria of *lactobacillus plantarum, lactobacillus buchneri, and lactobacillus rhamnosus. Lactobacillus buchneri* was added to enhance aerobic stability after opening the silos. The chemical additive was calcium carbonate^1,2,17,18^.

### Study design

#### Study design for silage preparation

The coffee pulp collected from the Gomma-2 coffee farm located 397 km South West of Addis Ababa and about 50 km West of Jimma town was stored in a deep freezer until required. Coffee pulp silage was prepared in 1-L laboratory-scale glass jars. Four treatments with three ensiling periods, each run in triplicate. Hence, a total of 36 bottles were used for the silage preparation. Biological silage additives were dissolved in sterilized tap water and 6.7 ml kg^–1^ fresh pulp was applied with a syringe. 13.8 gm/kg Carbonated lime was filled directly into the jar and manually mixed with the Silage. Subsequently, the whole pulps and additives were compacted with a compacting device to ensure a constant density. After sealing, the jars were stored in a tempered and dark room at a constant temperature of 20 °C. To preserve the sample at this temperature incubator was used. As shown in Table 1, the storage duration of 10, 20, and 40 days for all treatments was defined to determine the ingredient contents over time. The jars were weighed at the beginning and end of the storage periods to determine the quality of the Silage against parameters mainly on the lactic acid content of the silage materials. All treatments and storage durations were performed in triplicate.

**Table 1:** Different additives at different ensiling period (storage days).

Treatments	Storage days		
	10	20	40
HO	0.001 gm/kg	0.001 gm/kg	0.001 gm/kg
CaCO_3_	13.8 gm/kg	13.8 gm/kg	13.8 gm/kg
HO + CaCO_3_	Combination	Combination	Combination
Control	Without additives	Without additives	Without additives

#### Study design for biogas production from coffee pulp and pulp silage

The substrates (pulp and pulp silage) were analyzed in batch anaerobic digestion tests. The substrates were chopped with the help of a chopping machine. Before starting the process, 14 digesters starting from T1 − T14 were prepared. T14 was the inoculum that was taken from digestates of the previous batch of anaerobic tests. Tests were performed in 500 ml glass vessels using 375 ml inoculums and 16.67 gm substrate. The vessels were shaken once a day to resolve sediments and scum layers. Tests were conducted at 38 °C in a water bath. The Biogas produced was collected using glucose bags over a defined period of 60 days and measured weakly using a biogas analyzer. Methane, carbon dioxide, oxygen, and hydrogen sulfide content were determined at least eight times during the batch test. The inoculums without substrate were run as a control set in each case. The methane yields from the inoculums were subtracted from the total methane yields of digestion of a substrate mixture to inoculums to determine the actual methane yields of the substrate (i.e. pulp and pulp silage with different additives).

Methane yields were calculated as the sum of methane volume produced over 60 days with reference to the dry organic matter to the batch test (oDM added) or with reference to the original organic dry matter (oDM org), i.e. the amount of oDM before ensiling with respect to organic mass losses during storage.

### Physio-chemical property determination of coffee pulp and coffee pulp silage

#### Dry matter/total solid

The dry matter content of the coffee pulp and coffee pulp silage with different additives was determined by putting samples with known amounts of crucible weight and weighing samples by using an analytical balance. A triplicate of five grams of each sample for the precision of data was taken and weighed carefully. The weighed samples were placed inside the oven, and the temperature was maintained at 105 °C for 24 h^2,5,6^. After 24 h, the samples were taken out of the oven and put into desiccators to cool at room temperature without absorbing moisture. Finally, the cooled sample was weighed. The procedures are described below:

The empty porcelain crucible was dried in an oven overnight at 105 °C, and then, the crucible was cooled in desiccators to room temperature, weighed by high sensitive electronic balance, and recorded. After that, the 5 gm sample was weighed and put into the crucible, and the sample was put into the oven and dried overnight. After removal from the oven, the sample and crucible was cooled in desiccators to room temperature, then the oven-dry crucible plus sample was weighed and recorded the weight (W)^2,5,11^.

The percentage of dry matter in the sample was calculated as follows:

1 $$\mathrm{\%\,DM}=\frac{\mathrm{W}-\mathrm{Wc}}{\mathrm{Wo}-\mathrm{Wc}}\times 100,$$

where Wo = weight of sample plus crucible before drying, W = weight of dried sample + crucible, Wc = weight of the crucible, % D.M. = percentage of dry matter.

#### Moisture content

Five grams of Pulp and pulp silage with different additives were taken into a pre-dried crucible. After 24 h, the crucible with 5 gm sample was removed from the oven and put in desiccators until room temperature and weighed till constant weight was observed and the loss in weight of the pulp and pulp silage gave a percentage of the moisture content^1,5,6,18^.

#### Crude ash

Five gm of the pulp and pulp silage was placed in a porcelain crucible and transferred into a muffle furnace at a temperature of 550 °C for 5 h. The crucible was cooled in desiccators and weighed again^1,5^ then the weight loss was recorded as the ash content of the sample. The percent ash content (dry basis) was calculated.

#### Organic dry matter/volatile solid

The determination of volatile solids for the pulp and pulp silage was done using 105 °C dried samples. The procedure and working conditions were done like crude ash. Then, the volatile solid was calculated as follows:

2 $$\%\,VS=100-\mathrm{\%\, ash}.$$

#### Total nitrogen and crude protein

Nitrogen was estimated using the Kjeldhal method^3,5,6,8,11,16,18,23^ and the percentage of nitrogen was calculated using Eq. (3) and the value was converted to the percentage of protein by multiplying it with 6.25.

3 $$\mathrm{\%N}=\frac{\mathrm{volume \,\,of\,\, HCl \,\,in\,\, litre}\times \mathrm{N}.\mathrm{HCl}(0.1\mathrm{N})\times 14(\mathrm{mass\,\, of\,\, nitrogen})}{\mathrm{WS}}\times 100,$$

where WS = weight of sample in g, N. HCl = normality of hydrochloric acid.

#### Determination of pH

The pH of the raw pulp and the ensiled was determined by preparing a 1:10 ratio of sample to distilled water. For this 10 gm. of the sample was measured into the beaker, 100 ml of distilled water was added, homogenized, and mixed with the help of a magnetic stirrer for 15 min. The mixed sample was subjected to settle down, and pH was measured by a pH meter^1,5,16^.

#### Crude fat

The Soxhlet extraction techniques were used for the determination of fat content. 2 gm of the sample was taken and separately covered with a porous filter paper and put in a thimble. The thimble was then placed in a Soxhlet reflux flask and mounted into a weighed extraction flask containing 200 ml of petroleum ether. The upper end of the reflex flask was covered by a condenser. The process was continued for 4 h^9,16^. The solvent was recovered, and the flask was dried in the oven at 60 °C for 30 min, then cooled in desiccators and re-weighed to obtain the final weight of the oil extract (fat), which was then expressed as % of the fat of the sample. The percentage (%) of fat content was calculated using the following formula:

4 $$\mathrm{\%\,\, crude\,\, fat}=\frac{\mathrm{W}2-\mathrm{W}1}{\mathrm{Sample\,\, mass\,\, in \,\,gm}}\times 100,$$

where $\mathrm{W}1=$ Mass of flask, $\mathrm{W}2=\mathrm{mass \,\,of\,\, flask \,\,plus\,\, fat}$.

#### Determination of organic carbon content

The carbon content of the coffee pulp and pulp silage was estimated from the volatile solid or organic matter content of the samples. The following formula was applied to calculate the percentage composition of the carbon content^5^.

5 $$\mathrm{\%\,C}=\frac{\mathrm{\%\,VS}}{1.724}.$$

#### C/N ratio determination

Once the percentage of carbon and nitrogen in the pulp and pulp silage was determined. The carbon-to-nitrogen ratio (C/N) is simply calculated by dividing the percentage of carbon by the percentage of nitrogen^3,11^.

6 $$\frac{\mathrm{C}}{\mathrm{N}}=\frac{\mathrm{\%\,C}}{\mathrm{\%\,N}}.$$

#### Determination of coffee pulp in terms of minerals and some heavy metal contents

##### Minerals content determination

The coffee pulp was oven dried at 65 °C in the oven, ground, and passemd 1 mm mesh size. After homogenization of the sample, 1 gm. lithium Meta borate and 0.5 gm. lithium tetra borate were weighed in a platinum crucible, 0.2 gm of the finely ground sample was added to the crucible that contained the borate mixture, and it was mixed with stirring rod that is also made up with platinum crucible. The sample was fused in a muffle furnace for 45 min at 950 °C. The crucible and the melt were taken out from the muffle furnace using the crucible protective glove. The crucible and the content were put into a 400 ml beaker containing (1 + 19) HNO_3_ of 100 ml solution, then covered with a watch glass, heated the content, and tilted the crucible to help digestion. It was kept overnight, and on the next day, a Teflon coating stirring magnet was added to the crucible and put on a magnetic stirrer. It was stirred until complete dissolution. The solution was transferred into a 500 ml volumetric flask by means of a funnel. It was washed several times and diluted till the mark of the volumetric flask. After that, the content of calcium oxide, magnesium oxide, sodium oxide, potassium oxide, ferrous oxide, and manganese oxide was determined with the help of an Atomic Absorption Spectrophotometer (A.A.S.)^5,10^.

##### Heavy metal determination

0.25 gm of the sample (1 mm mesh size) was taken into a 100 ml beaker, and the content was digested with perchloric acid, hydrochloric acid, and nitric acid. The final solution was transferred to 50 ml volumetric flask; the flask was filled till the mark. Finally, the content of heavy metals was determined by AAS^3,11,24^.

### Biogas digester composition

#### Feedstock

The feedstock for the study was coffee pulp and coffee pulp silage. Similar amounts of inoculums were used in order to determine the Biogas of each feedstock. The effect of additives on biogas production in this study was evaluated.

### Experimental setup

#### Batch anaerobic digester

**Figure 1:**
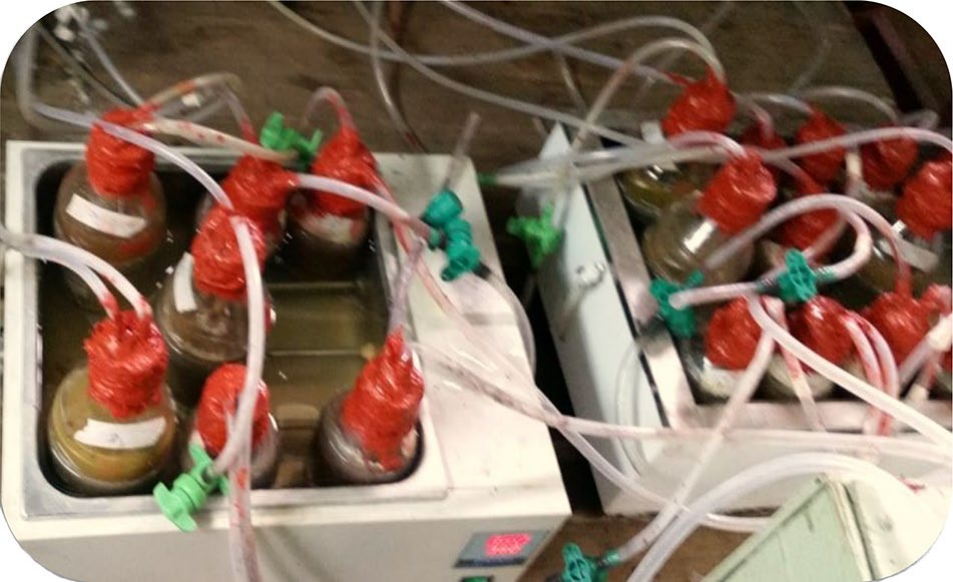
Batch anaerobic digester set up.

A 500 ml holding capacity bottle was used as a digester. To create the anaerobic condition, the bottles were covered by a rubber stopper with an inlet and outlet and sealed with a gas kit maker. The gas pipe with an 8 ml internal diameter of 0.5 and 1 m length was immersed into the digester, as it is shown in Fig. 1. The 0.5 m long hose was stretched up to the bottom of the solution to measure the pH of the slurry, while the 1 m long hose did not touch the solution and was used to collect the gases. The gases were collected by glucose bags to know the quality and quantity of the gas produced. Both houses were controlled by a valve. The pH of the slurries was measured in 14 days intervals. The temperature of the digester was fixed to 38 °C (mesophilic condition)^7,17,24^.

#### Biogas and its quality determination

The amounts (quantity) of Biogas from the digesters were collected by glucose bags and it was taken to a volumetrically calibrated vessel. The total Biogas produced for each treatment was done by deducting the value of the inoculums to know the value of each digester. For that matter, the total amount of Biogas produced from each digester was presented. The quality of Biogas (that is percentage of Methane) was measured by biogas analyzer within 8 days intervals until the gas production was ceased. The glucose bags that collect the Biogas was fitted to the gas analyzer and another glucose bag was used to collect the gases from the gas analyzer. After reading the quality of gases the bags which collect the gas was properly removed from the gas analyzer and closed by glucose bag cups, after that the total amount of gas was measured by graduated measuring syringes to know the total amount of gases collected^17,24^.

### Slurry analysis

#### Total solid and volatile solid determination

After the biogas production ceased, the total content and volatile content of the slurries for each treatment were measured to determine the solid reduction, that is, total solid (T.S.) and volatile solid (VS). The same methods used for the determination of feedstock were used for the determination of % T.S. and %VS of slurry.

#### Determination of the total nitrogen and Total phosphorus content of the slurry

The total nitrogen content of the slurry of each treatment was done by the persulfate digestion method (Method 10,071). To perform this work, first, the C.O.D. reactor was turned and heated until it reached 105 °C. Nitrogen persulfate reagent powder was added to each of the total hydroxide reagent vials, then 2 ml of sample was added to the vial, capped both vials, and shaken vigorously for at least 30 s. Then the vials were placed in the reactor and heated for 30 min. After 30 min, the vials were removed from the reactor and waited until cooled. Then the content of one total nitrogen reagent A powder was added to each vial. It was shaken for 15 s, 3 min reaction period was allowed, then Nitrogen reagent B powder was added, capped the vials, and shaken for 15 s. Then 2 ml of the digested sample was transferred to a total acid reagent vial, mixed, read the content of total nitrogen at the wavelength of 395 nm.

Total phosphorus (PhosVer 3 with Acid Persulfate Digestion Method): A pretreated diluted slurry sample with acid and persulfate was digested for 30 min in a HACH COD digester at 150 °C. A blank prepared from distilled water was treated and measured in a similar manner to the samples. Upon being treated with a PhosVer3 reagent powder pillow, the orthophosphate reacted with molybdate in an acid medium to produce a mixed phosphate/molybdate complex with an intense molybdenum blue color and measured at 880 nm.

### Data analysis

For the comparison and physicochemical analysis of the feedstocks, yield, and quality of Biogas, average values of the triplicate data were used. The Anova was performed using Microsoft Excel 2013, and the data was drawn using the Origin 8 software.

## Results and discussion

### Characteristics of coffee pulp and pulp silage

#### Minerals and some heavy metal contents of coffee pulp

Coffee pulp was characterized by the mineral contents like calcium oxide, potassium oxide, sodium oxide, magnesium oxide, iron oxide, and manganese oxide, and the heavy metals lead, chromium, and cadmium were analyzed. The results were indicated as follows:

Mineral and Heavy metal content of Coffee pulp.

CaO < 0.01%, Na_2_O = 1.02%, K_2_O = 3.60%, MgO = 0.22%, Fe_2_O_3_ = 0.52%, MnO < 0.01%, Pb, Cd and Cr < 0.005 ppm.

Minerals calcium and manganese and all the heavy metals were below the instrumental detection limit of 0.01% and 0.005 ppm, respectively. In contrast, the coffee pulp had a value of 1.02% Na_2_O, 3.60% K_2_O, 0.22% MgO and 0.52% Fe_2_O_3_. The results of the present study were lower than the study conducted by Solomon Demeke^1^, which showed the content of calcium, magnesium, and potassium at 0.5%, 0.13%, and 5.3%, respectively.

#### Protein and fat content analysis of the treatments

The feedstock’s protein and crude fat content were evaluated in Table 2 below. The crude fat content was in the range of 0.50 under control for all ensiling periods to 3.50 under biological additives (homo fermentative lactic acid bacteria) for ensiling periods 10, whereas; the protein content was in the range between 11.75 in the chemical and biological additives environment for the ensiling period of 40 days to 14.56 Silage with chemical additives at 10 day ensiling period.

**Table 2:** Percentage of crude fat and crude protein of the feed stock.

Descriptive of feed stocks	Treatments	Fat (%)	Protein (%)
Silage without additives at 10 day ensiling period (control)	T1	0.50	12.50
Silage with biological additives at10 day ensiling period	T2	3.50	12.69
Silage with chemical additives at10 day ensiling period	T3	1.00	14.56
Silage with both additives at 10 day ensiling period	T4	2.50	11.81
Silage without additives at 20 day ensiling period (control)	T5	0.50	14.44
Silage with biological additives at20 day ensiling period	T6	2.50	13.81
Silage with chemical additives at20 day ensiling period	T7	1.50	12.81
Silage with both additives at 20 day ensiling period	T8	1.00	13.00
Silage without additives at 10 day ensiling period (control)	T9	0.50	13.38
Silage with biological additives at10 day ensiling period	T10	3.00	14.13
Silage with chemical additives at10 day ensiling period	T11	1.00	13.63
Silage with both additives at 10 day ensiling period	T12	2.00	11.75
Raw coffee pulp	T13	3.00	12.69

#### Characteristics of coffee pulp and pulp silage

The Moisture content (%MC), dry matter (D.M.) or total solid (T.S.), organic dry matter (ODM) or Volatile solid (VS), ash (fixed solidly), and Organic carbon (O.C. %), total nitrogen (T.K.N. %) content of the feedstock were shown in Table 3. Standing from the results, it is possible to conclude that the feedstocks had a moisture content above 80%, which is the best input for biogas production. The ash content was below 20%, which indicates the organic content is higher than the inorganic content. As a result, the coffee pulp, as well as the ensiled one, is the best candidate for biogas production.

**Table 3:** Characteristics of the feed stock and inoculum.

Treatments	MC %	TS %	VS %	Ash %	OC %	TKN %
T1(C10)	83.88 ± 1.35	16.12 ± 1.35	91.91 ± 1.35	8.09 ± 0.50	53.31 ± 0.79	2.00 ± 0.04
T2(BA10)	82.53 ± 0.58	17.47 ± 0.58	91.82 ± 0.58	8.18 ± 1.05	53.26 ± 0.34	2.03 ± 0.02
T3(CA10)	82.75 ± 0.65	17.25 ± 0.65	87.29 ± 0.65	12.71 ± 2.15	50.63 ± 0.38	2.33 ± 0.04
T4(BA + CA)10	84.45 ± 0.66	15.55 ± 0.66	83.68 ± 0.66	16.09 ± 2.66	48.67 ± 0.38	1.89 ± 0.04
T5(C20)	83.44 ± 2.16	16.89 ± 2.16	91.61 ± 2.16	8.39 ± 0.19	53.14 ± 1.25	2.31 ± 0.03
T6(BA20)	81.55 ± 0.45	18.52 ± 0.45	92.55 ± 0.45	7.45 ± 1.10	53.68 ± 0.26	2.21 ± 0.03
T7(CA20)	80.38 ± 0.88	19.62 ± 0.88	85.05 ± 0.88	14.95 ± 0.47	49.33 ± 0.51	2.05 ± 0.04
T8(BA + CA)20	86.33 ± 0.44	13.67 ± 0.44	83.64 ± 0.44	16.36 ± 2.58	48.52 ± 0.26	2.08 ± 0.04
T9(C40)	87.70 ± 0.00	17.30 ± 0.00	90.61 ± 0.00	9.39 ± 0.00	52.56 ± 0.00	2.14 ± 0.04
T10(BA40)	83.33 ± 0.08	16.67 ± 0.08	90.77 ± 0.08	9.23 ± 0.99	52.65 ± 0.05	2.26 ± 0.03
T11(CA40)	81.27 ± 0.38	18.73 ± 0.38	85.27 ± 0.38	14.73 ± 0.70	49.46 ± 0.22	2.18 ± 0.03
T12(BA + CA)40	82.31 ± 0.05	17.69 ± 0.05	80.69 ± 0.05	19.31 ± 1.79	46.80 ± 0.03	1.88 ± 0.03
T13(Cp)	83.94 ± 0.67	10.42 ± 1.50	89.58 ± 1.50	15.62 ± 0.67	51.96 ± 0.87	2.03 ± 0.02
T14(In)	98.92 ± 0.04	48.14 ± 1.17	51.87 ± 1.17	1.08 ± 0.04	28.58 ± 0.68	1.40 ± 0.00

#### Silage pH

The pH value of the feedstock used in the treatment is shown below. The Silage produced without additives and biological additives was shown to lower pH in the range of 3.96 − 4.17. Similarly, the raw coffee pulp has also shown acidic pH = 4.80. On the contrary, the Silage with chemical and both chemical and biological additives has shown a neutral pH. The lower pH observed in the control and biological additives may attribute to the acid product. Whereas the neutral pH may attribute to the neutralization of the acid by the chemical additives (calcium carbonate additives). The pH of the raw pulp was evaluated by different researchers. Its pH value was 4.1. On the contrary, the present study was in agreement with the study conducted by. The pulp had a pH of 4.75. As a result, the pulp is acidic by nature.

Coffee pulp and pulp silage characteristics before anaerobic digestion.

T1 (pH = 4.12), T2 (pH = 3.96), T3 (pH = 7.46), T4 (pH = 7.33), T5 (pH = 4.17), T6(pH = 4.05), T7 (pH = 7.26), T8 (pH = 7.03), T9 (pH = 4.16), T10 (pH = 4.05), T11 (pH = 6.47), T12 (pH = 7.52) and T13 (pH = 4.80).

### The working conditions of the anaerobic digestion process

The biogas production and its quality were affected by the variables and conditions like temperature, pH, and C/N ratio. The results of this study are given below:

#### pH profile of the digester

The initial values of the digesters were T1 (6.88), T2 (6.63), T3 (7.21), T4 (7.19), T5 (6.92), T6 (7.02), T7 (7.15), T8 (7.03), T9 (7.02), T10 (6.88), T11 (6.80), T12 (6.89) and T13 (7.18). This pH is in agreement with the range of 6.25–7.5which is conducive for methanogenic bacteria to function in a proper way.

**Figure 2:**
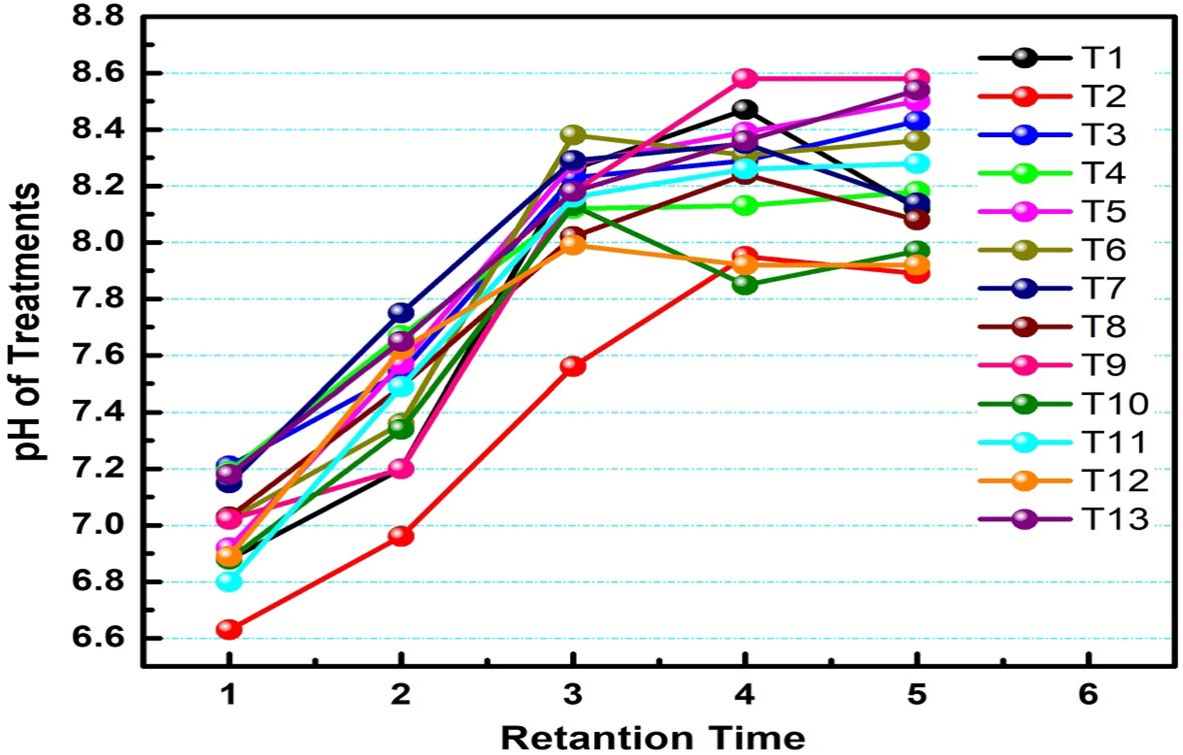
Average pH values of the digesters throughout the retention time.

From Fig. 2, the pH values of the thirteen treatments were determined, and their values were evaluated between two weeks intervals from the initial feeding time till the gas production ceased. In all cases, the pH increased from the feed time till the end of the digestion period, and the maximum pH was recorded by treatments T9 (8.58) and T13 (8.54) in the 8th week. From the Figure, it is possible to conclude the fermentation phase is already taking place in the ensiling period, and the microorganisms were performing their biogas production in a faster retention time. The result of ANOVA has shown that the pH of the digesters was not significant (p > 0.05). It showed that there is no relation between the pH of digesters.

#### Carbon to nitrogen ratio of the feedstock

From Table 4, the carbon-to-nitrogen ratios of the feedstock were between 20 and 27.This agrees with the value of 20:1 to 30:1 as recorded by as well as. This indicates that the raw coffee pulp, as well as the ensiled one by different additives, could serve as a feedstock for biogas production.

**Table 4:** C/N ratio of all the feed stocks.

Descriptive of the feed stocks	Treatments	C/N
Silage without additives at 10 days ensiling period (control)	T1	26.66
Silage with biological additives at10 days ensiling period	T2	26.24
Silage with chemical additives at10 days ensiling period	T3	21.73
Silage with both additives at 10 days ensiling period	T4	25.75
Silage without additives at 20 days ensiling period (control)	T5	23.00
Silage with biological additives at20 days ensiling period	T6	24.29
Silage with chemical additives at 20 days ensiling period	T7	24.06
Silage with both additives at 20 days ensiling period	T8	23.33
Silage without additives at 10 days ensiling period (control)	T9	24.56
Silage with biological additives at10 day ensiling period	T10	23.30
Silage with chemical additives at10 day ensiling period	T11	22.69
Silage with both additives at 10 days ensiling period	T12	24.89
Raw coffee pulp	T13	25.60
Inoculum	T14	20.42

### Amount and quality of Biogas produced by each digester at different additives with different ensiling periods

The biogas production potential of the feedstock was presented in terms of biogas yield and biogas quality (% methane). The average weekly biogas production, the cumulative Biogas, and the quality of Biogas produced by each digester are shown below.

#### Total biogas production

**Figure 3:**
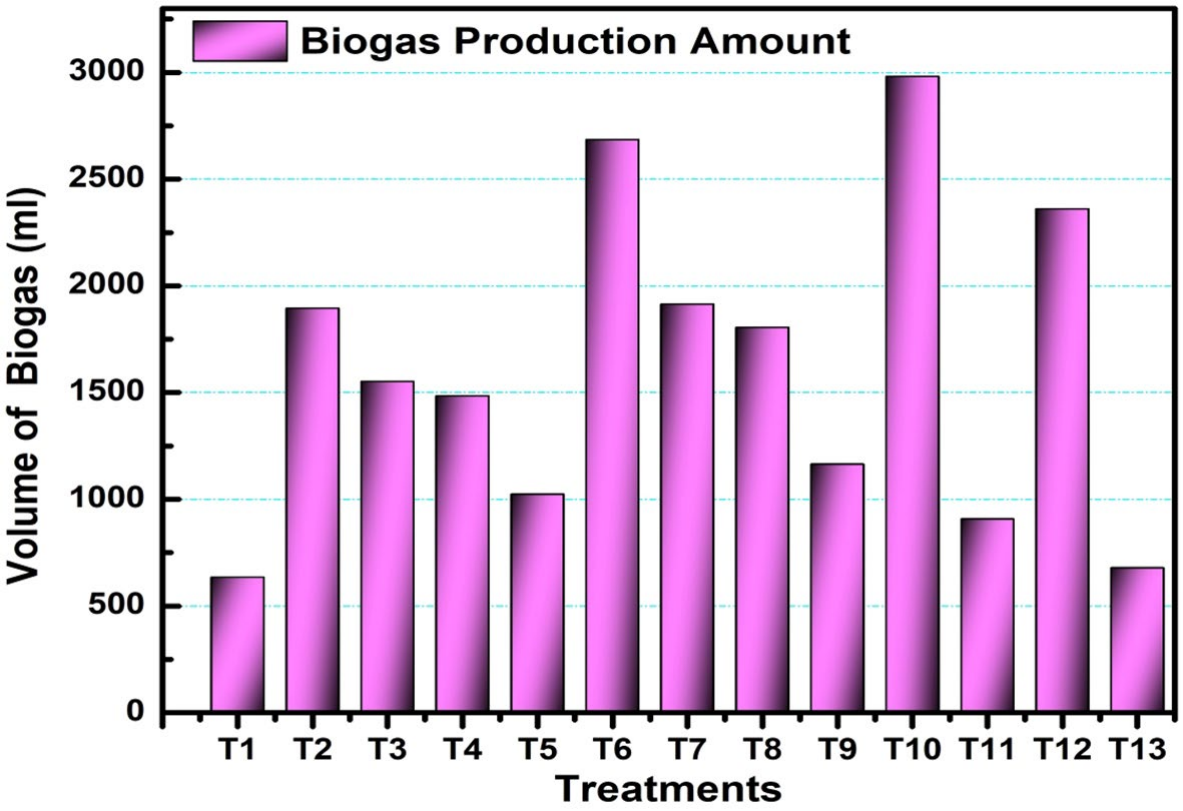
Comparison of total biogas production of digesters.

The total Biogas produced during the reaction period for all treatment digesters is presented in Fig. 3. It is clearly observed that T10 has higher productivity and T1 gives the lower one in terms of amount.

**Figure 4:**
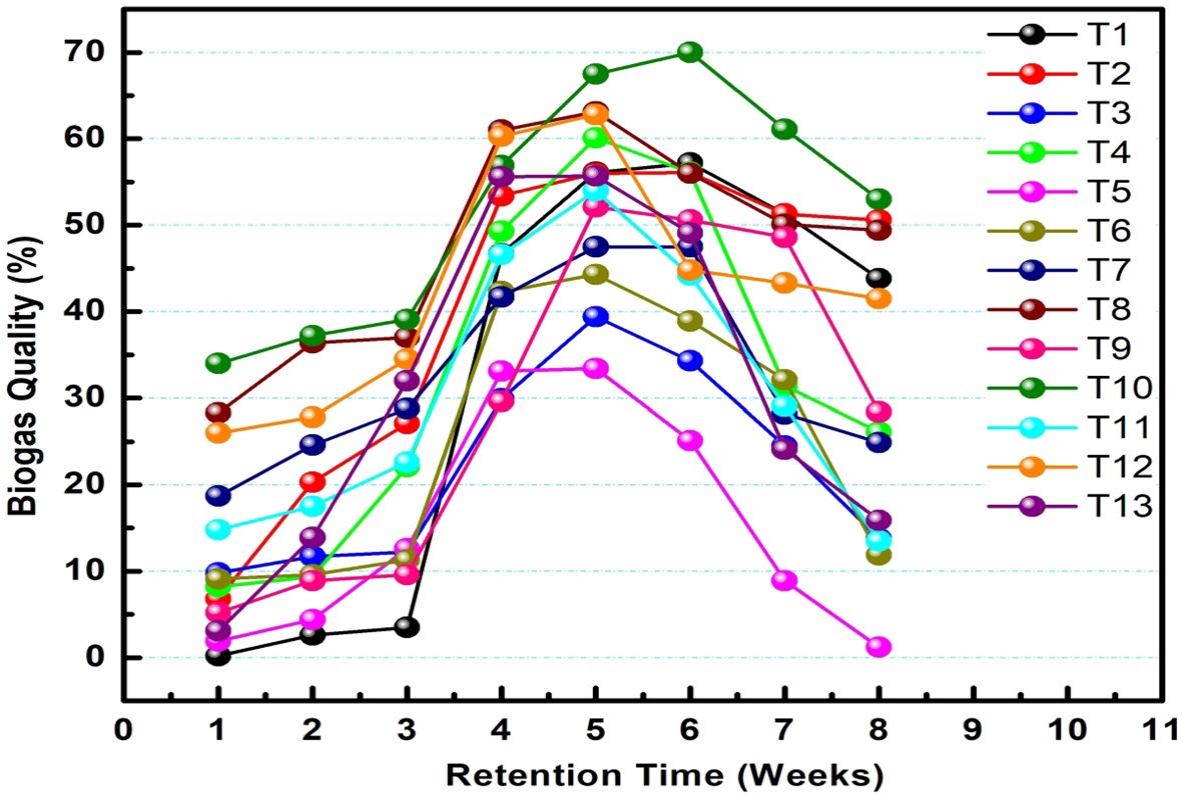
Weekly biogas quality of each digester throughout retention time.

As it is shown in Fig. 4, the quality of Biogas produced by each digester in the first, second, and third week of the digestion period was below 50%. This shows that the biogas quality below 50% is not combustible, and the other gases, like carbon dioxide, compared with other weeks, are higher than in these weeks. Therefore, the gas produced during this period needs to be discharged if it is not upgraded by cleaning the other gases like carbon dioxide by scrubbing it with lime, sodium hydroxide, or potassium hydroxide.

Treatments T3, T4, T5, and T6 continued to produce Biogas with a quality of less than 50% until the end of the reaction period. Such treatments produce a biogas quality of 34.3%, 33.4%, 44.6%, and 47.5% maximum value, respectively. After the fourth week, most of the treatments except the above four (T3, T4, T5, and T6) produced a total biogas quality of more than 50%. Which indicates the gases produced during this reaction period were combustible. In addition, depending on the system design and the type of feedstock, the biogas quality between 50 and 75% is pure Methane. After week six, the biogas quality was lowered, and finally, the production is all most ceased.

**Figure 5:**
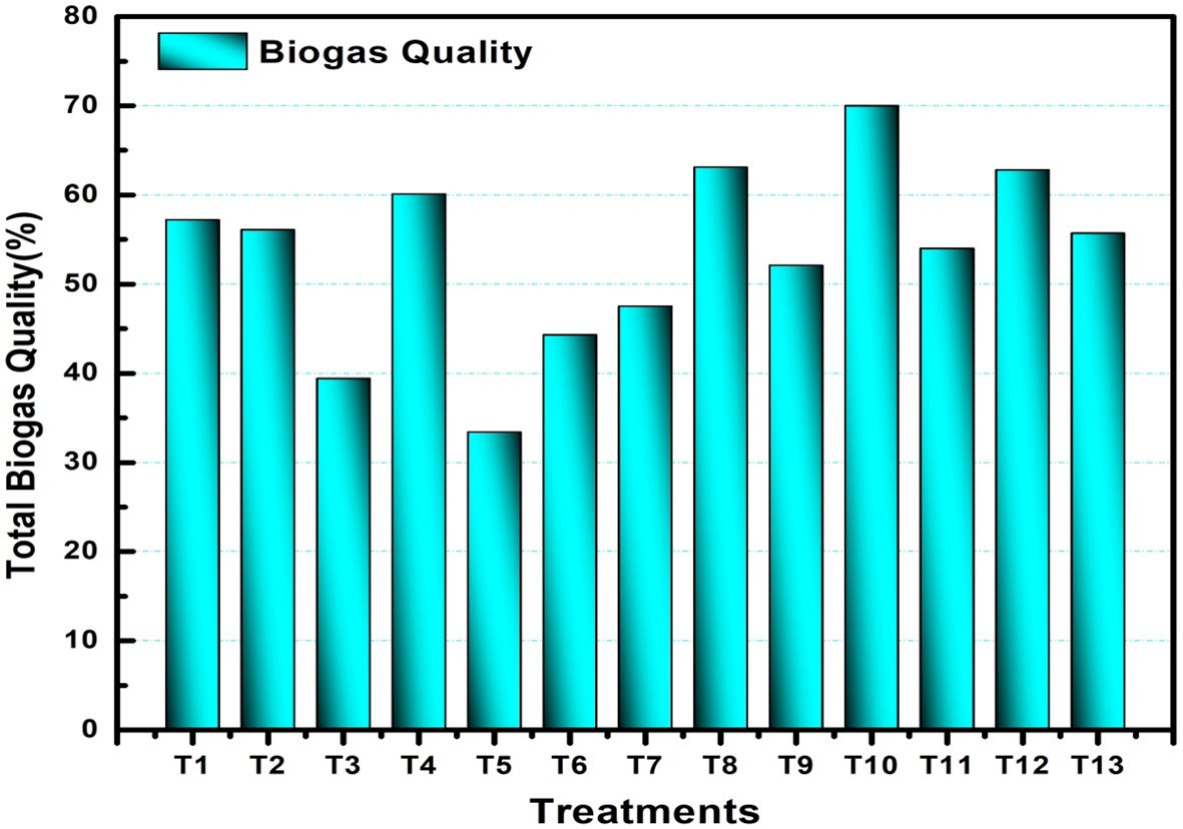
Total quality of biogas of each treatment (digester).

As shown in Fig. 5, the highest and lowest quality was produced by T10 (biological additives at 40 days ensiling period) and T5 (control at 20 days ensiling period), respectively. From the Figure, it was concluded that the T1, T5, and T9 are the controls ensiled at 10, 20, and 40 days; the biogas production was highest in the 10 days than the others. Biological additives with higher ensiling period positively affected the production of Biogas recorded. One-way ANOVA test results of treatments (digesters) between and within groups show that the p-value is less than 0.05, which indicates that there is a statistically significant difference between the volume of Biogas, percentage of Methane, and volume of Methane of all the treatments.

### Characteristics of the digester after digestion

#### Solid reduction after anaerobic digestion

Total solids and volatile solids of the feedstock for all of the digesters after digestion to determine the amount of solid reduced were analyzed. The total solid and the volatile solid of each digester before and after the digestion period were indicated in the Table 5 below.

**Table 5:** TS and VS reading of the treatments in the feeding time and after digestion.

Digesters	% TS initial	% VS initial	% TS final	% VS final	% reduction of TS	% reduction VS
T1	1.64	78.95	0.60	33.33	63.27	57.78
T2	14.22	96.97	0.80	0.00	94.40	100.00
T3	1.50	50.00	1.40	30.43	6.67	39.13
T4	1.98	50.85	0.60	0.00	69.85	100.00
T5	2.11	86.36	1.34	59.09	36.41	31.58
T6	3.84	97.78	0.84	0.00	78.03	100.00
T7	7.36	80.00	1.53	50.00	44.77	37.50
T8	14.81	57.50	1.11	0.00	92.50	100.00
T9	2.21	70.00	1.72	37.50	21.98	46.43
T10	12.50	85.00	5.56	0.00	55.53	100.00
T11	2.08	78.05	1.46	66.67	30.00	14.58
T12	5.16	86.00	0.68	0.00	86.80	100.00
T13	4.04	72.97	0.90	33.33	77.76	54.32
T14	2.87	50.00	0.75	0.00	73.83	100.00

Anaerobic treatment converts the organic pollutant into a small amount of sludge and a large amount of Biogas (methane and carbon dioxide). So, there must be a reduction of T.S. and VS in the conversion process. The high and low removal efficiency of T.S. (%) were seen for T2 (treatments with biological additives at 10 days ensiling period) and T3 (treatments with chemical additives at day 10 days ensiling period) and the low removal efficiency of VS was seen for T11 (treatment with chemical additives at 40 days ensiling period). The high values were observed at T2, T4, T6, T8, T10, T12, and T14 with the same value of 100% removal of VS.

The relatively higher removal efficiency of VS (%) than T.S. (%) was a very good indication of the high uptake rate of the organic fraction of total solid and the effectiveness of the anaerobic reactor in digesting the coffee pulp wastes the ensiling wastes of coffee waste at different ensiling periods with different additives under anaerobic digestion during the proper operating condition.

From the percentage reduction of total solids and volatile solids, it can be put forward that anaerobic digestion can reduce the amount and volume of the organic waste of coffee pulp which is disposed of in dumpsites. It can also reduce the cost of transport as well as the task of the municipality’s waste management system. Comparison of the volatile solid and total solid before and after anaerobic digestion gives an indication of the utilization of the organic content in the digester. Before digestion VS/TS ratio of the digesters was relatively higher before digestion than after digestion. This is an indication of the utilization of the organic components during anaerobic digestion.

### pH and nutrient values of the slurry

**Figure 6:**
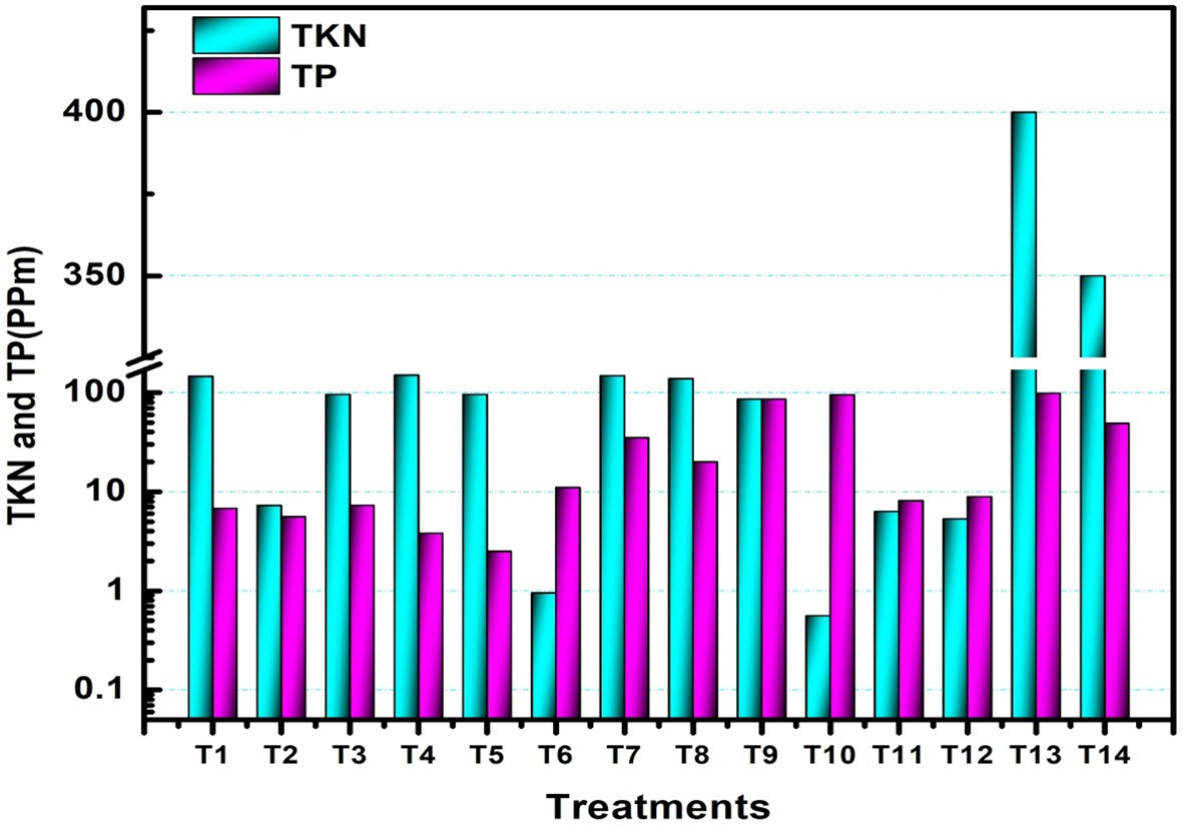
Total nitrogen and total phosphorus content of the slurry.

One advantage of anaerobic digestion is the use of the slurry as organic fertilizer. As a result, the pH and the macro-nutrients for the slurry of treatments, T1, T2, T3, T4, T5, T6, T7, T8, T9, T10, T11, T12 and T13 were determined, and its pH value was found that 8.12, 7.89, 8.43, 8.18, 8.5, 8.36, 8.14, 8.08, 8.58, 7.97, 8.28, 7.92 and 8.54, respectively. The values in the thirteen treatments were between the minimum and maximum accepted values of 6.0 and 8.5. The values of the macro-nutrients (total nitrogen and available phosphorus) are shown in Fig. 6 below:

As it can be seen from the Figure, the total nitrogen and total phosphorus content were highest for T13and its value was recorded at 400 ppm and 98.5 ppm, respectively. The minimum value was recorded at treatment (digester) T10 for total nitrogen content of 0.56 ppm. The minimum value of phosphorus was recorded in treatment (digester) T5, which was 2.5 ppm.

## Conclusion

This study suggests that coffee pulp may be used as a substrate for the production of Biogas in mesophilic environments. During the 40-day ensiling treatment period with the biological additives, more gas is produced in terms of quantity and quality. Additionally, more study is required to address various biological and chemical additives. Coffee producers will also benefit from using pulp waste to produce Biogas by treating it with biological additives. Therefore, they can benefit economically, socially, and healthily by avoiding the direct disposal of waste into the environment. The slurries are also used as natural fertilizers.

## Data Availability

The datasets used and/or analysed during the current study available from the corresponding author on reasonable request.

## Abbreviations

GHG: Greenhouse gas emission
CH_4_: Methane
CO_2_: Carbon dioxide
% DM: Percentage of dry matter
OC: Organic carbon
VS: Volatile solid
TS: Total solid
AAS: Atomic absorption spectrophotometer
COD: Chemical oxygen demand
T1: Control at 10 days ensiling
T2: Biological additives with 10 days ensiling
T3: Additive of calcium carbonate/chemical additives at 10 days ensiling
T4: Both chemical and biological additives at 10 days ensiling
T5: Control at 20 days ensiling
T6: Biological additive at 20 days ensiling
T7: Chemical additives at 20 days ensiling
T8: Both additives at 20 days ensiling
T9: Control at 40 days ensiling
T10: Biological additive at 40 days ensiling
T11: Calcium carbonate additive at 40 days ensiling
T12: Both chemical and biological additives at 40 days ensiling
T13: Raw pulp
T14: Inoculum
